# Role of Human Leukocyte Antigens at the Feto-Maternal Interface in Normal and Pathological Pregnancy: An Update

**DOI:** 10.3390/ijms21134756

**Published:** 2020-07-04

**Authors:** Chiara Tersigni, Federica Meli, Caterina Neri, Azzurra Iacoangeli, Rita Franco, Antonio Lanzone, Giovanni Scambia, Nicoletta Di Simone

**Affiliations:** 1 Dipartimento di Scienze della Salute della Donna, del Bambino e di Sanità Pubblica, U.O.C. di Ostetricia e Patologia Ostetrica, Fondazione Policlinico Universitario A. Gemelli IRCCS, 00168 Rome, Italy; caterina.neri@policlinicogemelli.it (C.N.); antonio.lanzone@policlinicogemelli.it (A.L.); nicoletta.disimone@policlinicogemelli.it (N.D.S.); 2 Facoltà di Medicina e Chirurgia A. A Gemelli, Università Cattolica del Sacro Cuore, 00168 Rome, Italy; meli.federica@gmail.com (F.M.); azzurra.iacoangeli1@gmail.com (A.I.); rita.f7593@gmail.com (R.F.); giovanni.scambia@policlinicogemelli.it (G.S.); 3 Dipartimento di Scienze della Salute della Donna, del Bambino e di Sanità Pubblica, U.O.C. di Ginecologia Oncologica, Fondazione Policlinico Universitario A. Gemelli IRCCS, 00168 Rome, Italy

**Keywords:** Human Leukocyte Antigen (HLA), Major Histocompatibility Complex (MHC), pregnancy, maternal–fetal interface, syncytiotrophoblast, pre-eclampsia.

## Abstract

The successful maternal tolerance of the semi-allogeneic fetus provides an apparent immunologic paradox. Indeed, deep invasion of placental trophoblast cells into maternal uterine tissue and the following growth of the fetus have to be tolerated by a pregnant woman’s immune system. Among the various possible protective mechanisms that may be involved in human pregnancy, the expression of a non-classical pattern of human leukocyte antigen (HLA) class I molecules and the complete lack of expression of HLA class II molecules in placental tissues seem to be the most relevant mechanisms of fetal escape from maternal immune recognition. The importance of HLA molecules in fetal toleration by the maternal immune system is highlighted by pregnancy complications occurring in cases of abnormal HLA molecule expression at the maternal–fetal interface. In this review, we summarize evidences about the role of placental HLA molecules in normal and pathological pregnancies.

## 1. Introduction

The successful maternal tolerance of the semi-allogeneic fetus provides an apparent immunologic paradox. Human pregnancy is commonly considered a nature’s allograft, but it is actually the placenta, not the fetus, which constitutes the transplant.

To avoid immunological recognition and rejection of fetal-derived tissues by the maternal immune system, three main mechanisms of immune escape occur in human pregnancy: (a) anatomical separation between mother and fetus tissues through the placenta; (b) modification of Human Leukocyte Antigen (HLA) molecule expression in the placenta; (c) suppression of maternal cell-mediated immunity.

In this full literature review, we describe the physiologic modification of HLA molecule profiles in the human placenta required for a successful normal pregnancy. Suppression of HLA molecule transcription in placental tissues prevents classical T-cell responses to fetal antigens. Furthermore, fetal somatic cells that fully express HLA molecules are normally anatomically separated from the maternal immune system by the placental trophoblast barrier, preventing direct contact and, thus, fetal antigens presentation to maternal T cells. As an additional protective mechanism to the fetus, maternal cellular immunity undergoes a hypo-reactive shift. Any defects in the reciprocal immunological adaptation between the fetus and the mother may compromise placental development and fetus growth. Particularly, it has been suggested that complete failure of these mechanisms would cause miscarriage, while partial failure of fetal immune tolerance could cause poor placentation and dysfunctional utero-placental perfusion [1,2]. Here, we particularly discuss the potential pathogenic role of abnormal placental expression of HLA molecules in the occurrence of obstetrical complications.

## 2. Methods

Using PubMed and Google Scholar databases, we performed comprehensive literature searches in the English language for studies on the expression and function of HLA antigens in both normal pregnancy and main obstetric disorders (miscarriage, preterm delivery, pre-eclampsia). We performed a categorical review of the literature by using the following search terms: “Human Leukocyte Antigen”, “Major Histocompatibility Complex”, “maternal-fetal interface”, “recurrent pregnancy loss”, “preterm delivery” “pre-eclampsia” and combinations of these. Articles from 1990 to 2020 were screened for relevance, validity and quality.

## 3. Mechanisms of Fetal Immune Escape from Maternal Recognition

### 3.1. Anatomy of Maternal–Fetal Interface

**Figure 1:**
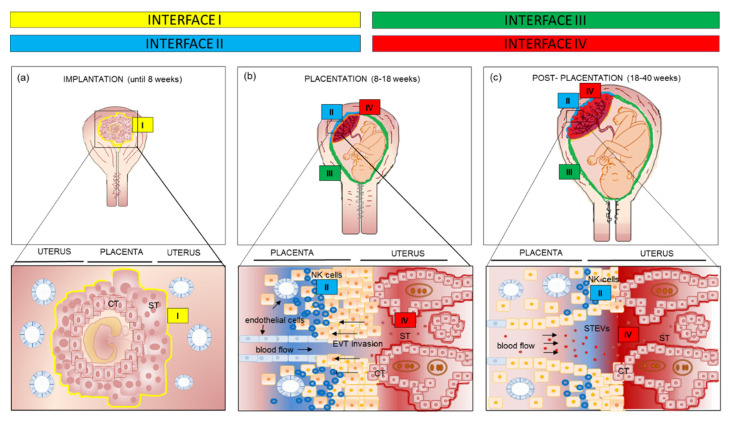
Main immunological interfaces between fetus and mother according to different gestational ages, from implantation to placentation process and post-placentation period. (**a**) Until 8 weeks of gestation the interface I is represented by ST invading the decidua; (**b**) in the second trimester of pregnancy the interface I is replaced by the interface II (decidual natural killer cells—extravillous trophoblast), III (decidua parietalis—amniochorion) and IV (maternal blood—ST); (**c**) in the third trimester interface IV is the dominant one. CT: cytotrophoblast; ST: syncytiotrophoblast; EVT: extravillous trophoblast.

Semi-allogeneic trophoblast forms several interfaces with the maternal immune system depending on gestational age and anatomic location [1] (Figure 1). In the early stage of implantation, the blastocyst is surrounded by trophectoderm that will develop into both the definitive villous placenta, constituted by cytotrophoblast (CT) and syncytiotrophoblast cells (ST), as well as the invading extravillous trophoblast cells (EVT) that invade into the uterus to tap into the maternal blood supply.

The earliest interface (interface I) comprises ST of the implanting embryo and the uterine decidua. This is the only time that ST lies at a tissue interface. Chorionic villi are bathed directly in uterine gland secretions allowing a histotrophic nutrition [3] (Figure 1a) until the establishment of the intervillous circulation after 8 weeks.

During the second trimester, three new interfaces replace the interface I: (i) interface II between invading extravillous trophoblast (EVT) and maternal immune cells in the decidua; (ii) interface III between decidua parietalis and amniotic membranes; (iii) interface IV between the ST and maternal blood. ST represents the epithelial subtype surrounding chorionic villi and shows a large surface area directly in contact with the maternal blood (Figure 1b).

It is worth noting that ST continuously shed extracellular vesicles (EVs) into intervillous space and, thus, into maternal circulation. EVs are membrane bound, cell-derived particles released as part of both normal physiological functions—such as hemostatic regulation and cell maintenance—and as a consequence of cellular stress (such as in pre-eclampsia) [2,4]. The term EV encompasses exosomes (~100 nm in diameter), microvesicles (0.1–1 μm) and apoptotic bodies (0.5–5 μm) [2,4]. ST-derived extracellular vesicles (STEVs) released into intervillous spaces are collected into venous blood in the uterine veins, go back into the heart, pass through the maternal pulmonary capillary bed and out into the systemic circulation. In normal pregnancy, STEVs are released constitutively and are thought to have a role in inducing maternal adaptive changes, which include suppressed maternal cell mediated immunity (see more below) [4]. Indeed, STEVs contain a complex cargo of RNAs, proteins, glycans and lipids, travelling from the apical surface of ST to target cells of the maternal body, and are considered conveyors of biological massage between the mother and the fetus. It is commonly agreed that STEVs released into maternal circulation represent an extension of interface IV.

In the first half of pregnancy, interfaces I and II dominate, and this is where the success of the pregnancy is determined early [2,5]. Interface II diminishes after 16 weeks while interface IV is activated with the onset of the uteroplacental circulation at 8–9 weeks [5] and enlarges with placental growth to become the dominant interface after 20 weeks (Figure 1c) [6,7]. Furthermore, STEVs shedding into the maternal circulation progressively increases from the first to the third trimester of pregnancy, contributing to the dominance of the interface IV in the second half of pregnancy.

### 3.2. Peculiar Expression of HLA Class I and II in the Human Placenta

The Human Leukocyte Antigen (HLA) system is a gene complex encoding the Major Histocompatibility Complex (MHC) proteins, or HLA molecules, in humans. These cell-surface proteins are also called human transplantation antigens and are responsible for the regulation of the immune system in humans [8].

**Figure 2:**
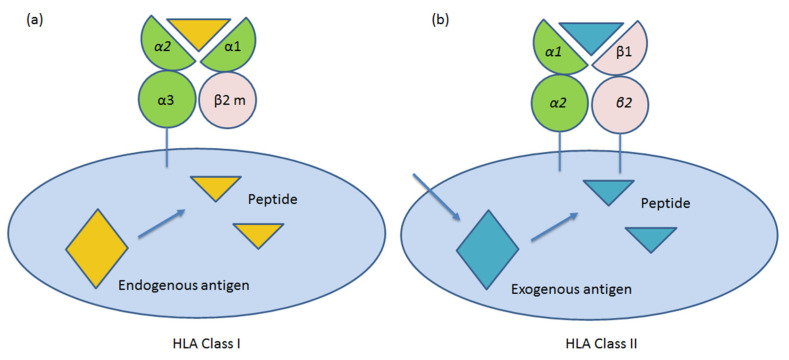
Schematic representation of molecular structure of human leukocyte antigen (HLA) class I (**a**) and II (**b**) molecules in human cells. β2 m: β2 microglobulin.

HLAs class I (A, B and C) molecules (Ia) consist of a glycosylated heavy chain and a light chain β2-microglobulin. The heavy chain has three extracellular domains (α1, α2 and α3), a transmembrane region and an intracytoplasmic domain. The α1 and α2 domains contain variable amino acid sequences, and these domains determine the antigenic specificities of the HLA class I molecules (Figure 2a). Class I HLA molecules are expressed on the cell surface of all nucleated eukaryotic cells. They also occur on platelets, but not on red blood cells. Their role is to protect “self” healthy cells from immune cells recognition. According to the “missing self” hypothesis, the absence or altered expression of HLA class I molecules renders target cells susceptible to NK cell attack [9]. Furthermore, another function of HLA class Ia molecules is to display peptide fragments of proteins from within the cell (damaged or infected) to cytotoxic T cells; this will trigger an immediate response from the immune system against not-self antigens [10].

On the other hand, HLA class II molecules (DP, DM, DO, DQ and DR) are heterodimers of two non-covalently associated glycosylated polypeptide chains: α and β (Figure 2b). The α and β chains are transmembrane and have the same overall structures. An extracellular portion composed of two domains (α1 and α2, or β1 and β2) is anchored on the membrane. Polymorphisms of class II molecules occur in the first amino terminal β1 domain Figure 2a.

HLA class II molecules are expressed constitutively on professional antigen-presenting cells (APC), such as dendritic cells, mononuclear phagocytes and B cells. The antigens presented by class II peptides are of extracellular origin and are loaded onto HLA class II molecules by phagocytosis. These antigens stimulate the proliferation of T-helper cells, which in turn elicit B-cells to secrete antigen-specific antibodies [9]. In particular, activation of T cells requires two signals, a first signal, which is antigen-specific, is provided through the T cell receptor (TCR) which interacts with peptide-HLA class II molecules on the membrane of APC. A second signal, the co-stimulatory signal, is antigen nonspecific and is provided by the interaction between co-stimulatory molecules expressed on the membrane of APC (i.e., CD80 and CD86) and the T cell (i.e., CD28).

In human pregnancy, tight control of human leukocyte antigens (HLA) class I and II expression in chorionic and EVT is essential for successful outcome [11].

In humans, the villous ST does not express any HLA class I or class II molecules [12] so that T cells are theoretically not able to recognize and bind to the main placental barrier. This “antigens hiding” is clearly a highly effective mechanism to protect the placenta from maternal immune rejection. In contrast, the EVT only expresses HLA class I and not class II molecules, thus, it does not act as an APC and cannot initiate direct allo-recognition to maternal CD4+ T cells [5,6].

Although trophoblasts have been demonstrated to contain the IFN-γ receptor and the necessary transduction mechanism, they do not express HLA class II antigens, even after IFN-γ exposure [13].

The lack of HLA-A and HLA-B, and general lack of HLA class II expression on EVT prevents maternal T cell allo-immune responses against paternal-derived HLA antigens. Furthermore, the only expression of the classical HLA-C and of the atypical class I antigens (Ib), such as HLA-G and HLA-E molecules, is thought to protect EVT from destruction by decidual natural killer (dNK) and T cells [14] (Table 1).

**Figure 3:**
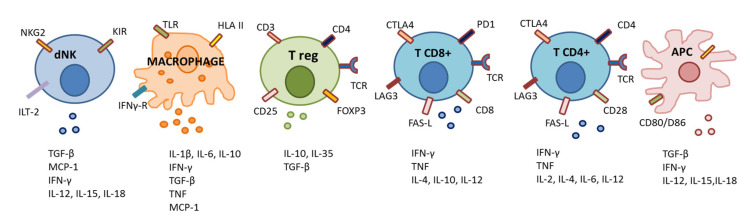
Main immune cells involved in immune tolerance at feto-maternal interface. APC: antigen presenting cell; CTLA4: cytotoxic T-lymphocyte associated protein-4; dNK: decidual natural killer cell; HLA: human leukocyte antigen; IL: interleukin; ILT-2: immunoglobulin-like transcript 2 receptor; IFN: interferon; KIR: killer cell Ig-like receptors; LAG3; lymphocyte activation gene-3; MCP: monocyte chemoattractant protein; NKG2: natural killer group 2 receptor; PD1: programmed cell death 1; TCR: T cell receptor; TGF: transforming growth factor; TLR: toll-like receptor; TNF: tumor necrosis factor.

As EVT invades maternal uterine tissues during the implantation process, 80% of maternal leukocytes are represented by dNK, 10% by macrophages and, for the remaining 10%, by T cells (Figure 3). However, these proportions vary deeply together with gestational age. At term pregnancy, T cells are the predominant lymphocyte population within the maternal decidua (40–70% of CD45+ leukocytes), although also dNK (20–50%) and decidual macrophages (10–15%) are present in relatively high amounts [15].

Thus, during the first half of pregnancy, NKs are relatively abundant in maternal decidua compared to other maternal immune cells. dNKs show a tolerogenic phenotype due to the loss of NK cytotoxic activity and the acquisition of immunomodulatory properties. In particular, low cytotoxicity in dNK cells can be explained by their particular receptor expression profile. They express the killer cell immunoglobulin-like receptors (KIR, which identify polymorphic HLA-C), the natural killer group 2 receptor (NKG2, which recognizes HLA–E), the immunoglobulin-like transcript 2 receptor (ILT-2, which recognizes HLA-G) and [16].

HLA-C is a central molecule in the NK and T cells’ immune activity. In particular, it has been shown that levels of HLA-C expression on the cell surface influence the efficacy of cellular immune responses to viral-, allo- and self-antigens [15]. In particular, the differences in HLA-C restricted allo-responses or anti-viral responses have been suggested to influence pregnancy outcome. NK cells specifically recognize two groups of HLA-C allotypes, HLA-C1 and HLA-C2. They differ in the amino acid substitutions at position 80 of the HLA-C heavy chain (HLA-C1 has an asparagine while HLA-C2 has a lysine) [15]. The expression profile of killer cell Ig-like receptors (KIRs) gives dNK the ability to specifically recognize on the EVT the HLA-C1, utilizing the inhibitory KIR2DL2/3 receptors, or HLA-C2, using the inhibitory KIR2DL1 and the activating KIR2DS1 receptors [5,15,17]. In conclusion, the interactions of dNK with invading EVT differ in each pregnancy and result by the combination of maternal KIR haplotypes, maternal and fetal HLA-C allotype and NK cell education.

In addition to HLAC–KIR interaction at immunological interface II (NK-EVT), human trophoblast has been shown to adopt another mechanism of inhibition of dNK cytotoxicity. Particularly, it is known that the cytotoxic activity of NK cells is commonly induced by the interaction of the NK cell receptor NKG2D with its ligands, namely the MHC class I chain-related (MIC) proteins A and B and the UL-16 binding proteins (ULBPs). This receptor–ligand interaction activates a perforin-mediated cytotoxic pathway leading to the elimination of damaged and/or infected cells. Interestingly, in human placenta MIC ad ULBPs expression is constitutive, and these proteins are released in the soluble form by STEVs during pregnancy [17]. Placenta-derived soluble MIC and ULBPs have been shown to down-regulate the NKG2D receptor, and indirectly down-regulate the NKG2D-mediated cytotoxicity, on peripheral blood mononuclear cells (PBMC) and purified NK, CD8+, and γδ T cells [18,19].

Additionally, maternal decidual CD8+ T cells directly recognize allogenic HLA-C molecules of paternal origin. This immunological cross-talk is a key process in the development of the immune tolerance of the fetus during pregnancy. Interestingly, decidual CD8+ T cells show high expression of the co-inhibitory molecules Cytotoxic T-Lymphocyte Associated Protein-4 (CTLA4), Programmed Cell Death 1 (PD1) and Lymphocyte Activation Gene-3 (LAG3) and low expression of cytolytic molecules. This uterine microenvironment is functional to inhibit CD8+ effector T cell activity and to maintain placental tolerance [20]. However, it has been shown that decidual CD8+ T cells retain the ability to respond to pro-inflammatory events since, upon stimulation in vitro, they produce interferon-γ, tumor necrosis factor-α, perforin and granzyme B [20].

Additionally, maternal regulatory T cells (Treg) play a pivotal role in establishing immune tolerance to invading EVT and preventing inflammatory responses to placental antigens [15,21]. Indeed, Tregs are highly represented in the human decidua [21]. It is extraordinary how in vitro co-culture of naïve CD4+ T cells with EVT, directly increased the proportion of T cells spontaneously differentiating into CD4+ FOXP3+ Tregs, [22,23]. Intriguingly, some authors have proposed that Treg differentiation would occur after specific recognition of fetal HLA-C by maternal T cells [15] but the precise mechanisms are still a matter of investigation.

In conclusion, we can summarize that the HLA-C molecule has a central role in modulating the activity of the main factors of the cell-mediated maternal tolerance to the fetus: NK, CD8+ and Treg cells.

In contrast to a classical HLA-C, that is, a class Ia molecule, which is highly polymorphic, with a high number of alleles encoding a high number of functional proteins, HLA class Ib molecules display a very low polymorphism, with a small number of alleles encoding a limited number of proteins. Similarly, to HLA Ia molecules, HLA Ib molecules can also bind peptides generated through a proteasome-driven degradation of cytosolic proteins and present them to specific subpopulations of CD8+ T cells [14]. The main function of these molecules is to modulate the immune responses in both physiological and pathological conditions [24]. In particular, HLA-G is monomorphic and forms a homodimer that can bind with high avidity to LILRB1, an inhibitory receptor expressed by all decidual APC, to KIR2DL4 expressed by NK cells [25] and to CD160 expressed by T lymphocytes, NK cells and endothelial cells [26]. These interactions deviate immune responses towards a tolerogenic rather than an immunogenic response. In particular, upon interaction with these receptors, HLA-G (i) inhibits T cells proliferation and cytotoxicity [27] and elicit the expansion of regulatory T cells [28], (ii) inhibits differentiation, proliferation and cytokine production in B lymphocytes [29], (iii) inhibits proliferation and cytotoxicity of peripheral blood and uterine NK cells [30], (iv) inhibits chemotaxis of different T, B and NK cell populations by downregulating chemokine receptors expression on their surface [31] and (v) inhibits phagocytosis and production of reactive oxygen species in neutrophils [32]. This extraordinary and selective immunological interaction between mother and fetus has the added benefit of only occurring when there is direct physical contact between HLA-G+ EVT and uterine APC, allowing maternal APC elsewhere in her body to function normally [15]. Finally, NK–EVT interactions involve HLA-E and NKG2A/C as well as HLA-F and KIR3DS1 interactions that may lead to degranulation and release of perforin (PRF) and granzymes (GZMs) [15], eliciting immunomodulatory effects that are still a matter of investigation.

### 3.3. Other Mechanism of Modulation of Maternal Cell-Mediated Immunity

Suppression of maternal cell-mediated immunity towards placental antigens, whether fetal-derived antigens-presentation might erroneously occur, is an additional, and not secondary, mechanism of immune tolerization at the maternal–fetal interface.

Induction of apoptosis of immune cells is one of the main mechanisms involved in the negative regulation of T cell proliferation. Interestingly, it has been shown that the human placenta is able to release bioactive Fas ligand (FasL) carried by STEVs able to perform apoptosis of maternal T cells, thus promoting the fetal allograft survival [33].

In addition, trophoblast cells sustain the maternal–fetal tolerance by secreting various chemokines and cytokines. In particular, trophoblast cells selectively recruit peripheral NK and T cells to the decidua via the secretion of a strictly controlled cascade of chemokines such as CXCL12, CXCL16 and CCL3 [34]. Furthermore, trophoblast cells are responsible for an additional mechanism of immune modulation at the feto-maternal interface mediated by the release of G-CSF, IL-10 and TGF-β. Indeed, these cytokines induce: (i) the shift of the Th1/Th2 ratio toward Th2; (ii) the inhibition of pro-inflammatory Th17 [21].

Recently, it has been demonstrated that immunosuppressive cytokine IL-35 is constitutively expressed in human first-trimester trophoblasts and acts by inhibiting the proliferation of human naive T cells and converting them into IL-35-producing induced regulatory T cells (iTR35) in an IL-35-dependent manner through phosphorylation of STAT1 and STAT3 [35]. In vivo studies confirmed that mice with immunologically spontaneous abortions have lower levels of IL-35 and iTR35 cells at the maternal–fetal interface, and neutralizing anti-IL-35 antibodies enhances abortion rates [35]. In conclusion, trophoblast-secreted cytokines make a pivotal contribution to the establishment of a tolerogenic milieu at the fetal–maternal interface and are responsible for the inhibition of maternal cell-mediated immunity to fetal antigens.

## 4. Abnormal Expression of Placental HLA Molecules in Obstetric Syndromes

Inadequate maternal tolerance of EVT has been proposed as a possible immunological trigger of insufficient trophoblast invasion of the maternal uterus. According to the extent of the defective tolerization towards the semi-allogeneic fetus by the maternal immune system, subsequent miscarriage or pre-eclampsia may occur [2,36,37].

Before 8 weeks, the spiral arteries are plugged by CT and there is no intervillous perfusion. Hypoxic environment establishing at the implantation site is functional to the neo-angiogenetic process. Between 8 and 12 weeks the arteries progressively unplug starting the intervillous circulation and, thus, the change from histotrophic to haematochorial placenta. When trophoblast invasion of the placenta bed is inadequate, intervillous perfusion occurs prematurely. Then, either miscarriage ensues or pregnancy continues with dysfunctional placental perfusion which leads to pre-eclampsia (PE) [37].

### 4.1. Miscarriage

Spontaneous miscarriage occurs in about 15–20% of all clinically recognized pregnancies [38]. Recurrent miscarriage (RM), defined by three consecutive pregnancy losses prior to 23 weeks of gestational age, is an obstetrical syndrome that shows a heterogeneous etiology and has a prevalence of 3–5% of all fertile women [38]. In about 40% of cases of RM no causes can be found and RM is defined as unexplained. Abnormal placental expression of HLA molecules has been proposed as a mechanism involved in the pathogenesis of unexplained RM.

Reduced expression of HLA-G has been observed in miscarriage tissues collected from women with a history of RM [39]. In particular, in vitro studies on JEG-3 cells have suggested that over-expression of miR-133a may be responsible for decreased HLA-G expression, providing evidence that miR-133a regulates HLA-G expression by reducing translation and could be involved in the pathogenesis of unexplained RM [39]. Consistently, HLA-G was found to be increased in term placentas of women with a previous history of RM compared to women without previous adverse pregnancy outcomes [40]. On the other hand, in a recent meta-analysis, no differences in terms of uterine NK levels have been found between women with RM and normal pregnancy outcomes [41].

The importance of the proper expression of the fetal HLA-C molecule for a successful pregnancy outcome has been indirectly suggested by the evidence that HLA-C antibodies could be involved in the pathogenesis of unexplained RM [42].

As an additional immunological anomaly responsible for RM, decreased proportions of decidual Tregs have been reported in cases of unexplained RM compared to miscarriage with karyotype abnormalities [43] as well as in PE compared normal pregnancies [44,45].

Abnormal expression of HLA-DR in CT and ST from miscarriages [46] and in ST of villitis areas obtained from term placentae [47] have also been reported.

These observations support the evidence that proper trophoblast expression of both HLA class I and II molecules is essential for a good pregnancy outcome. Aberrant trophoblast expression of HLA molecules early in pregnancy might represent an additional mechanism involved in the pathogenesis of unexplained miscarriage.

### 4.2. Pre-Eclampsia

PE is a relatively common disorder, complicating about 3–5% of all pregnancies. It is still a major cause of severe maternal and newborn morbidity and mortality worldwide [48]. It is defined by the occurrence of blood hypertension and substantial proteinuria after the 20th week of gestation. PE is probably heterogeneous in origin as it is in presentation. According to the “three stage model” of the development of PE [2], at the time of conception and implantation of the embryo, reduced immune tolerance of paternal-derived fetal antigens could occur (first stage) for some mechanisms that are still unclear. Between the 8th and 18th weeks of gestation, inadequate EVT invasion into the placental bed follows, determining inadequate remodeling of spiral arteries and poor placentation. Insufficient placental development may become clinically relevant at different gestational ages, according to the severity of the defect, leading to the third stage of the syndrome, when an insufficient maternal blood supply of the fetal–placental unit occurs [2]. Then, an oxidatively stressed and dysfunctional placenta releases factors (such as Flt-1, Endoglin and STEVs) into the maternal circulation which causes a generalized systemic inflammatory response, hypercoagulability and endothelial dysfunction.

Therefore, a reduced tolerance of placental antigens by the maternal immune system is believed to constitute the primum movens in the etiopathogenesis of PE, but the precise immunological defects occurring early in pregnancy are still unknown.

It has been shown that the type of combination of maternal KIR with fetal HLA-C genes influences the risk of PE and reproductive success [49]. In particular, it seems that women with dNK lacking activating KIR (KIR-AA genotype) when interfacing with a fetus expressing HLA-C2, are at an increased risk of developing PE, although subsequent studies have not confirmed this observation [50].

In a recent study, we have shown aberrant expression of HLA-DR in STEVs obtained by dual placental perfusion only from patients with PE but not from normal pregnancies [51]. Immunohistochemistry examination confirmed HLA-DR positivity on the apical surface of the ST in placentae from PE subjects [51]. Interestingly, the ST is the epithelium that releases circulating STEVs and constitutes the feto-maternal interface I, in the first trimester, and interface IV, in the second half of pregnancy. Consistently with our observation, abnormal HLA-DR expression in placentae from women with PE, particularly, in endovascular CT of inadequately remodeled utero-placental arteries has also been shown [52].

The transcriptional coactivator CIITA is required for HLA-D expression. In human trophoblast CIITA has been shown to be repressed epigenetically, likely as an effect of hypermethylation [53]. A mechanism implicating non-coding RNAs has also been suggested [54]. Intriguingly, the transfection of trophoblast cells with CIITA expression vectors induces the expression of HLA class II molecules [55]. However, the exact mechanisms of the regulation of HLA-D expression in the placenta are still a matter of investigation. Nonetheless, intracellular expression in primary CT has been previously demonstrated, and in most human cellular types HLA-D expression has been shown to be inducible in vitro by interferon-γ [56].

Our main hypothesis is that the PE defective suppression of HLA-DR might occur in the ST cells during early implantation. If HLA-DRs were of fetal origin, they would be highly immunogenic towards the maternal immune system. This would be likely to negatively interfere with the tolerization process in the maternal–fetal interface and cause a defective placentation that would later reveal clinically as overt PE. It is also likely that the oxidative stress and tissue inflammation occurring in the placenta of PE women might induce HLA-D aberrant expression later, in the third stage of the syndrome. In this case fetal HLA-D molecules, whether able to process and present to maternal immune cell fetal-derived antigens, could contribute to amplifying a systemic inflammatory response in the mother. Finally, it cannot be excluded that aberrant expression of HLA-D molecules in the ST might have a role in all three stages of the disease, involving different mechanisms of the maternal immune response.

Currently available data do not allow to draw any definite conclusions. More studies are required: (i) to confirm placental HLA-DR expression in a larger number of pre-eclamptic compared to healthy pregnant subjects; (ii) to confirm whether the HLA-DR is of fetal origin; (iii) to define its mode of synthesis in the ST; (iv) to evaluate the immunogenicity of HLA-DR positive STEVs.

### 4.3. Preterm Delivery

Datema and co-workers have specifically investigated whether the abnormal placental expression of HLA class I or II molecules might occur in pregnancy complicated by spontaneous preterm delivery (PTD). They did not observe differences in the expression of HLA on chorionic and extravillous cytotrophoblasts in the cases of PTD analyzed compared to normal pregnant women. However, higher expression levels of HLA class Ia molecules in amnion epithelial cells in PTD has been reported [14]. More recently, increased expression of HLA-G molecules has been shown in the placental sections of women with PTD compared to normal pregnancy [57]. Further studies are needed to clarify whether (a) a different pattern of placental HLA expression might be associated with PTD; (b) a different placental HLA expression might be pathogenically involved in the occurrence of PTD.

### 4.4. Others

Recently, other lines of investigation have proposed a pivotal role for HLA-C allo-recognition by dNK in the delicate process of limiting EVT invasion and preventing deep invasion and placentation that is associated with the placenta accrete spectrum. Indeed, those conditions are associated with abnormal adherence of the placental trophoblasts to the uterine myometrium, which can lead to maternal fatal bleeding at delivery [58].

As a whole, these observations suggest that failure of one or more mechanisms allowing fetus immune tolerance could be mainly responsible for the development of pregnancy complications.

## 5. Discussion

Among the immunological protective mechanisms that may be involved in successful pregnancy, the complete suppression of HLA class II molecules in both CT and ST cells and the unique expression of a non-classical pattern of HLA class I molecules seems to be relevant and crucial. This extraordinary modulation of HLA molecule expression on trophoblast explains how successful pregnancy is possible, only because both the female immune system and the placenta undergo deep changes of protein expression. Any defect of this elegant and complex process of reciprocal immunological adaptation between the fetus and the mother might potentially translate into an obstetric failure.

## Figures and Tables

**Table 1:** Expression of HLA class I and II molecules at feto-maternal interface.

	HLA-A, -B	HLA-C	HLA-G	HLA-E	HLA-DP	HLA-DQ	HLA-DR
Fetal tissues ^1^	+	+	−	+	+	+	+
ST ^2^	−	−	−	−	−	−	−
VT	−	−	−	−	−	−	−
EVT	−	+	+	+	−	−	−

^1^ no direct contact with maternal blood and tissues. ^2^ direct contact with maternal blood and tissues. ST: syncytiotrophoblast; VT: villous trophoblast; EVT: extravillous trophoblast.

## Abbreviations

CT	Cytotrophoblast
ST	Syncytiotrophoblast
EVT	Extravillous trophoblast
HLA	Human Leukocyte Antigen
MHC	Major Histocompatibility Complex
dNK	Decidual natural killer cells
EVs	Extracellular vesicles
STEVs	Syncytiotrophoblast-derived extracellular vesicles
PE	Pre-eclampsia
KIRs	Killer cell Ig-like receptors
